# Clinical utility of hepatitis C virus core antigen (HCVcAg) assay to identify active HCV infection in hemodialysis and renal transplant patients

**DOI:** 10.1371/journal.pone.0250263

**Published:** 2021-04-22

**Authors:** Suresh Ponnuvel, Gnanadurai John Fletcher, Raghavendran Anantharam, Santosh Varughese, Vinoy George David, Priya Abraham

**Affiliations:** 1 Department of Clinical Virology, Christian Medical College, Vellore, Tamil Nadu, India; 2 Department of Nephrology, Christian Medical College, Vellore, Tamil Nadu, India; National Taiwan University Hospital, TAIWAN

## Abstract

**Background:**

The prevalence of HCV infection is high and it is a major cause of liver-related morbidity and mortality in hemodialysis and renal transplant patients. Diagnosis of hepatitis C virus (HCV) infection requires both HCV antibody screening and confirmatory nucleic acid testing (NAT). Hepatitis C virus core antigen (HCVcAg) is a reliable direct viral marker to identify active HCV infection.

**Aim:**

To assess the clinical utility of HCV core antigen to identify active HCV infection in hemodialysis and renal transplant patients.

**Methods:**

A representative total of 231 plasma samples with a predominance of low viral load were included for HCVcAg testing and its performance characteristics were compared with the gold standard HCV RNA.

**Results:**

Comparison of HCVcAg with HCV RNA showed an excellent specificity of 99% (95% CI: 94.7 to 100%) and sensitivity of 80.62% (95% CI: 73.59 to 87.7%). Likewise, the PPV and NPV of HCVcAg were 99.1% (95% CI: 93.7% to 99.9%) and 80.2% (95% CI: 74% to 85.2%) respectively. The correlation between HCVcAg and HCV RNA was found to be good (R^2^ = 0.86, p<0.0001). Among common Indian HCV genotypes (1, 3 & 4), good correlation was observed between HCV RNA and HCVcAg (R^2^ = 0.81, p <0.0001).

**Conclusions:**

It is the first Indian study to show that HCVcAg is a reliable, cost-effective direct marker to identify active HCV infection in hemodialysis and renal transplant patients. Implementation of HCVcAg testing could improve the accessibility to efficacious and affordable disease management in hemodialysis and renal transplant patients. In HCVcAg negative cases, sequential testing with anti-HCV antibody followed by HCV RNA could be a reliable and cost-effective approach.

## Introduction

Globally, 80 million people are living with chronic hepatitis C virus (HCV) infection [1]. HCV infection is a significant cause of liver-related morbidity and mortality among hemodialysis and kidney transplant patients. The global prevalence of HCV infection in end-stage renal disease (ESRD) patients is 7.5% [2]. The serum HCV positivity in kidney transplant (KT) recipients is 10-fold higher than the general population [3]. In India, based on the anti-HCV and HCV RNA, the prevalence of HCV infection among hemodialysis patients is 4.3% to 45% and 27.7% respectively [4,5].

Patients with chronic kidney disease (CKD) are at high risk of acquiring HCV infection. Several high-risk factors have been identified for HCV infection among dialysis patients, which include prolonged vascular exposure, multiple blood transfusions, duration of end-stage kidney disease, mode of dialysis, and concurrent prevalence of HCV infection in the dialysis unit. Chronic HCV infection causes impaired health, increased risk of liver disease, higher cardiovascular mortality, and cryoglobulinemic syndrome in patients with hemodialysis [6,7]. In renal transplant recipients, HCV infection is independently associated with acute rejection, chronic allograft nephropathy, diabetes, and de novo glomerulonephritis [8,9].

The diagnosis of HCV infection is traditionally based on the detection of anti-HCV antibody and HCV RNA. However, the HCV antibody cannot be detected in the window period of 70 days [10]. Further, anti-HCV testing has not been reliable in dialysis patients because of the blunted humoral immune response that occurs with renal disease. The diagnostic limitations of antibody detection are that it cannot distinguish between current or past infection, and the false-negative rate is 17.9% in hemodialysis patients [11]. Thus, reliable direct markers such as HCV RNA or possibly HCV core antigen (HCVcAg) are needed to diagnose active HCV infection.

HCV RNA quantification is the recommended assay for the diagnosis, evaluation, and treatment by Kidney Disease Improving Global Outcomes (KDIGO) clinical practice guidelines [12]. HCV RNA is also used to assess treatment adherence and sustained virological response at 12 or 24 weeks after treatment completion. HCV RNA quantification by real-time reverse transcription-polymerase chain reaction (RT-PCR) is highly sensitive, specific, and reliable but expensive, time-consuming, and requires sophisticated equipment and trained personnel [13]. Though HCV RNA quantification is widely accepted as a gold standard, it is not suitable for routine screening among hemodialysis patients in resource-limited settings. Recently, HCVcAg quantification assay was developed, and its clinical sensitivity of 90% is comparable to HCV RNA. This assay was proved useful for the detection of early and active HCV infections in patients undergoing dialysis.

HCVcAg represents a robust stable marker of active infection than HCV RNA. It is released into the plasma during viral assembly, and it can be detected a few days after HCV RNA [14]. It is more stable than HCV RNA at room temperature, allowing easy transportation [15]. A positive HCVcAg result confirms the viral replication activity. In this study, we have ascertained the clinical utility of HCVcAg assay as a reliable, cost-effective, and high throughput alternative to HCV RNA for the diagnosis of active HCV infection in hemodialysis and renal transplant patients.

## Methods

### Study design and patients

Ten mL of K_2_EDTA blood samples were collected from hemodialysis and renal transplant recipients. Blood samples were centrifuged at 2500 rpm for 10 minutes for plasma separation and stored at -80°C. Written informed consent was obtained from each patient before testing. We performed a study on 231 archived plasma samples from hemodialysis and renal transplant patients between January 2014 and December 2019. This study design was approved by the Institutional review board and ethics committee (IRB minute no: 12009) of the Christian Medical College, Vellore, India.

### Anti-HCV antibody estimation

Anti-HCV antibody was estimated by using chemiluminescent microparticle immunoassay (ARCHITECT HCV Ab, Abbott, Wiesbaden, Germany). The samples with <1.0 S/CO were considered negative. Samples with ≥1.0 to 5.0 S/CO and ≥5.0 S/CO were considered as weak positive and positive respectively. The weak positive samples were repeated in duplicates according to the testing manual.

### HCV RNA quantification and HCV genotyping

HCV RNA was quantified from plasma samples by using automated real-time PCR (Abbott Real-Time HCV RNA assay; Abbott Molecular, USA) with the dynamic range of quantification of 12 to 10^8^ IU/mL.

HCV RNA was extracted from plasma samples using QIAamp Viral RNA mini kit (Qiagen, Germany) as per the manufacturer’s protocol. Sequencing of the HCV NS5b region was done using hemi-nested PCR. First-round PCR was performed using Titanium® One-Step RT-PCR kit followed by second-round PCR was performed using Super Therm Taq DNA polymerase (Medox^®^). The amplified 392 bp product from the second-round PCR was sequenced after a clean-up step to remove primer dimers and excess dNTPs. The nucleotide sequences obtained were subjected to BLAST using the HCV BLAST sequence database. The primer sequences are shown in **Table 1.**

**Table 1:** Primer sequences.

PCR	Primer name	Primer sequences (5’→ 3’)	Target region
*First round PCR	Forward P1204	GGAGGGGCGGAATACCTGGTCATAGCCTCCGTGAA	NS5b
	Reverse P1203	GGGTTCTCGTATGATACCCGCTGCTTTGACTC	
*Second round PCR & *Sequencing PCR	Forward P1204	GGAGGGGCGGAATACCTGGTCATAGCCTCCGTGAA	
	Reverse NS5b IP	TGATACCCGCTGCTTTGACTCNACNGTCAC	

*Daniel et al. [16].

### HCVcAg quantification

HCVcAg (ARCHITECT HCV Ag, Abbott, Wiesbaden, Germany) quantification was performed from the stored plasma samples. Plasma samples were centrifuged at 7000 rpm for 7 minutes and transferred (200 μL) into 2 mL sample cups to avoid any potential debris blocking the Architect sample aspiration needle.The HCVcAg assay is a 2 step chemiluminescent microparticle immunoassay (CMIA) using microparticles coated with monoclonal anti-HCV for the quantification of HCVcAg. The first step of pretreatment lyses the viral particles and extracts the HCVcAg. In the second step, any HCV core antigen present in the pretreated sample binds to the anti-HCV coated microparticles conjugated with acridinium-labeled anti-HCV conjugate. The resulting chemiluminescent reaction is measured as relative light units (RLUs). The concentration of HCVcAg in each specimen was determined using an Architect HCV Ag calibration curve generated during the assay calibration. The linear range of HCVcAg quantification spans between 3.0 fmol/L and 20000 fmol/L. Samples with a concentration of >20000 fmol/L were diluted with automated dilution protocol. The assay cut-off threshold for a positive result was ≥3.0 fmol/L, whereas values of 3.0 to 10 fmol/L and >10 fmol/L were reported as weak positive and positive respectively. The weak positive samples (3.0 to 10 fmol/L) were repeated in duplicates as per the recommendation of the testing manual.

### Statistical methods

The sensitivity, specificity, positive predictive value (PPV), negative predictive value (NPV), and correlation (Spearman’s rank correlation) of HCVcAg were calculated with the gold standard HCV RNA using MedCalc^®^ v14.8.1 (Belgium) and GraphPad Prism software v8.0 (California). A p-value of <0.05 was considered statistically significant.

## Results

**Fig 1:**
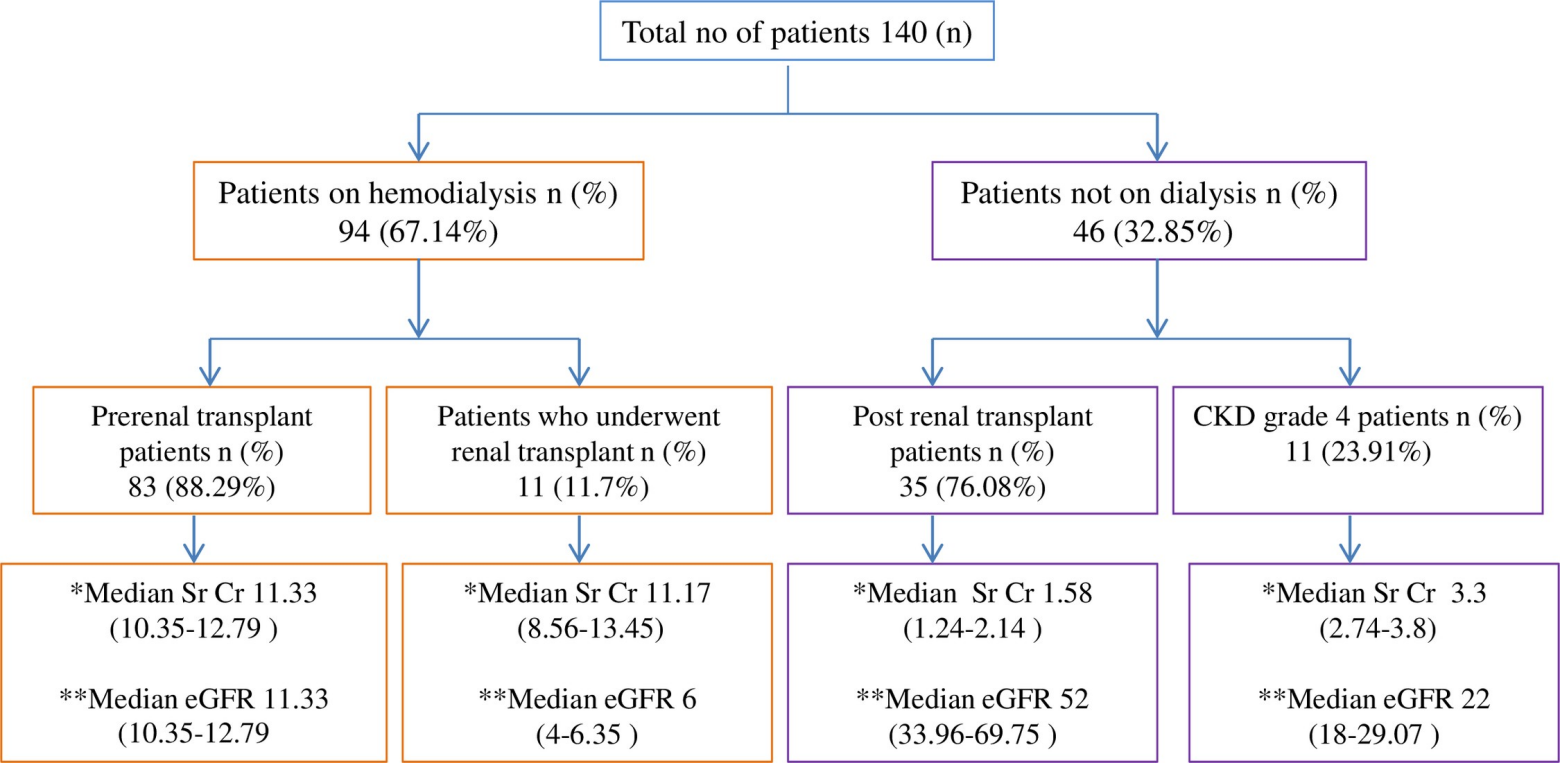
Patients classification with renal function profiles. *Median estimated glomerular filtration rate (eGFR) in ml/min/1.73m^2^ with 95% confidence interval (p <0.0001). **Median serum creatinine (Sr Cr) in mg/dL with 95% confidence interval (p <0.0001).

The HCVcAg quantification results of 231 plasma samples were compared with the gold standard HCV RNA for the calculation of sensitivity, specificity, correlation, PPV, and NPV. The demographic characteristics of enrolled patients are shown in **Table 2** and the renal function profiles are shown in **Fig 1.**

**Table 2:** Demographic characteristics of 140 enrolled patients.

Variables	n (%)
Median age (95% CI)	36 (34–38)
Male	106 (75.71%)
Female	34 (24.2%)
HCV/HBV co-infected	8 (5.71%)
HCV/HIV co-infected	2 (1.42%)
HBV infected	8 (5.71%)
HIV infected	2 (1.42%)
Intravenous drug users	2 (1.42%)
Liver transplant patients	2 (1.42%)

Abbreviations: n, number; CI, confidence interval; HCV, hepatitis C virus; HBV, hepatitis B virus; HIV, human immunodeficiency virus.

**Fig 2:**
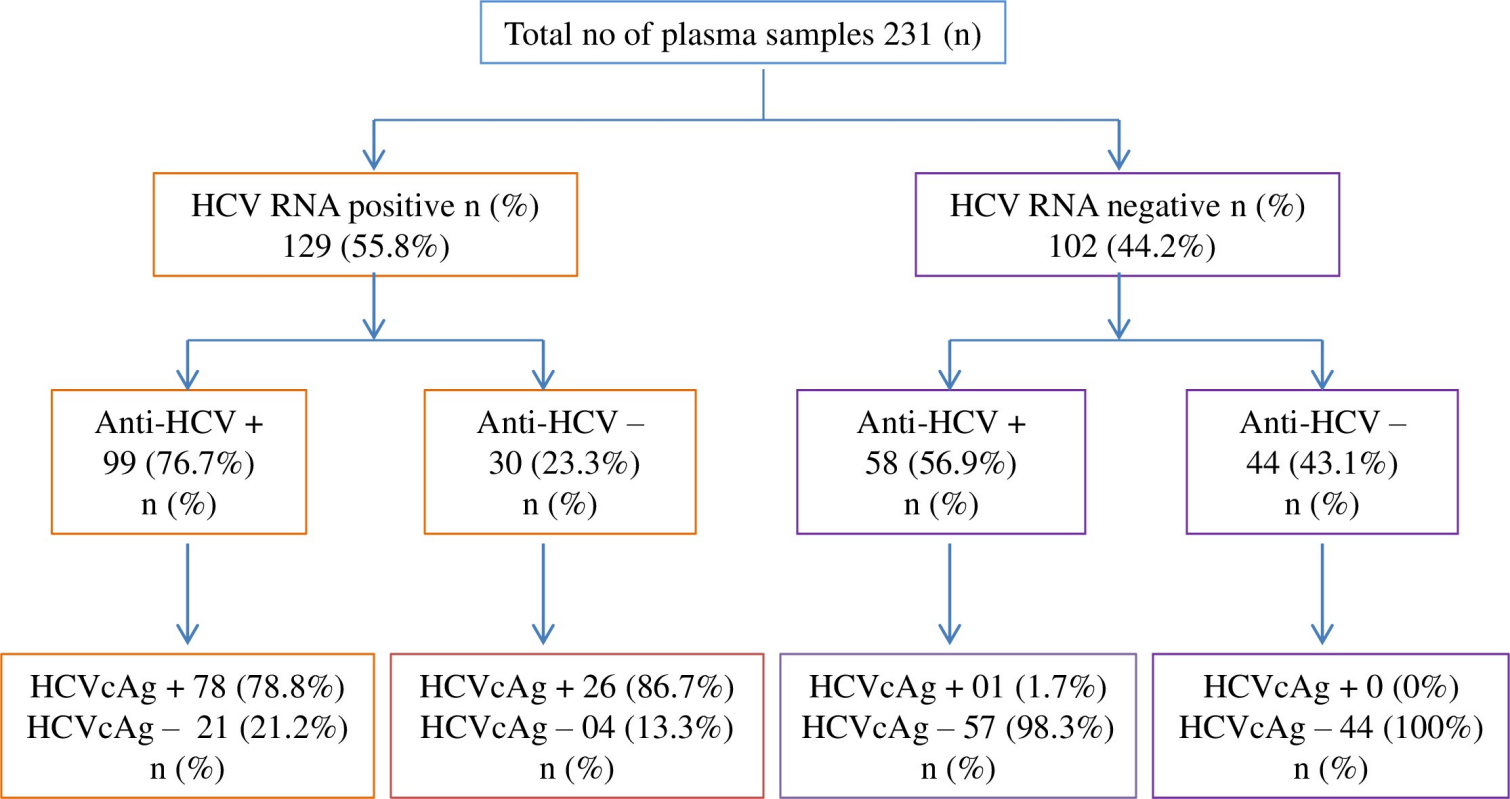
Classification of samples based on assay results.

Among the 231, 129 (55.8%) samples were positive for HCV RNA and 102 (44.2%) samples were negative for HCV RNA. Among 129 HCV RNA positive samples, 33 (25.6%) samples had a lower viral load of < 3 log IU/mL. In the 129 HCV RNA positive samples, 99 (76.7%) samples were anti-HCV positive, and the remaining 30 (23.3%) samples were anti-HCV negative. In the 99 anti-HCV positive samples, 78 (78.8%) samples were positive for HCVcAg, and 21(21.2%) samples were negative for HCVcAg. Likewise, in the 30 anti-HCV negative samples, 26 (86.7%) samples were HCVcAg positive and 4 (13.3%) samples were HCVcAg negative. Among the 102 HCV RNA negative samples, 58 (56.9%) samples were anti-HCV positive and the remaining 44 (43.1%) samples were anti-HCV negative. In the 58 anti-HCV positive samples, 1 (1.7%) sample was positive for HCVcAg, and 57 (98.3%) samples were negative for HCVcAg. In the 44 anti-HCV negative samples, all the 44 (100%) samples were negative for HCVcAg **(Fig 2).**

**Fig 3:**
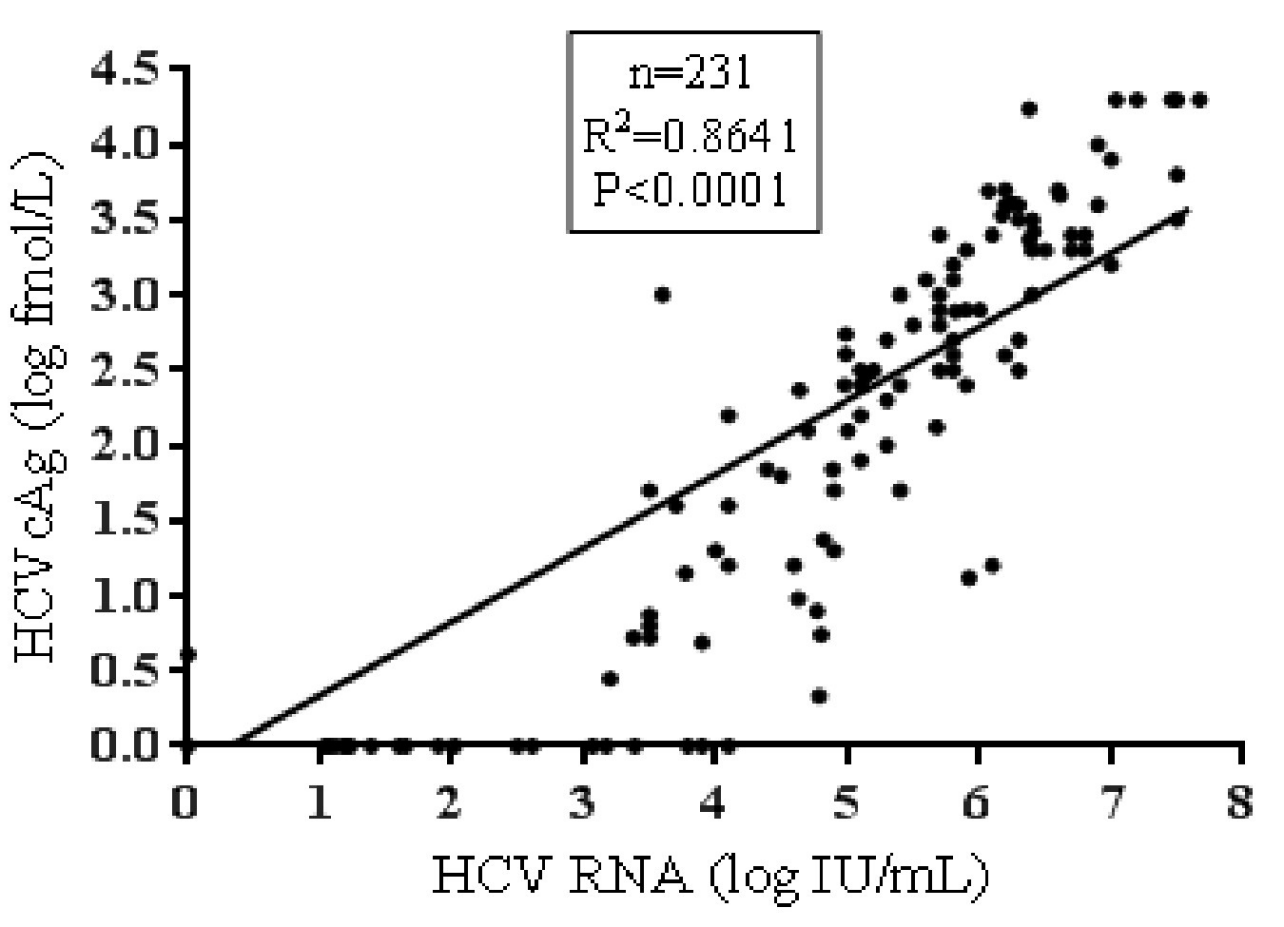
Overall correlation between HCV RNA and HCVcAg.

The overall sensitivity, specificity, PPV and NPV of the HCVcAg were 80.62% (95% CI: 73.59 to 87.7%), 99% (95% CI: 94.7 to 100%), 99.1% (95% CI: 93.7% to 99.9%) and 80.2% (95% CI: 74% to 85.2%) respectively. The overall correlation between HCV RNA and HCVcAg was good (R^2^ = 0.86, p<0.0001). The median HCV RNA was 1.9 log IU/mL (95% CI: 0 to 3.76 IU/mL), and the median HCVcAg concentration was 0 log fmol/L (95% CI: 0 to 0.73) **(Fig 3)**.

**Fig 4:**
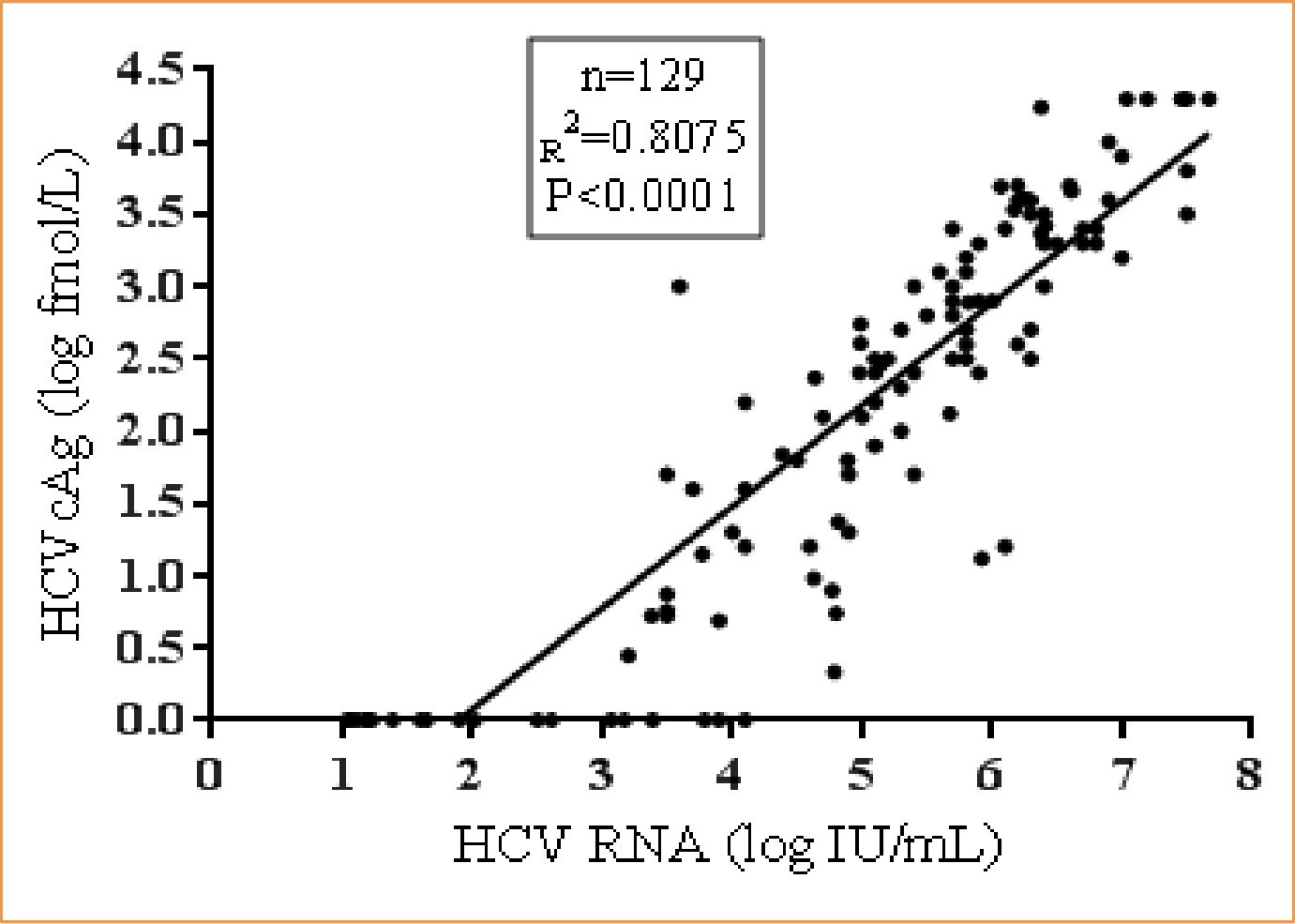
Correlation between HCV RNA and HCVcAg in HCV RNA positive samples.

In the HCV RNA positive samples (n = 129), a good correlation was found between HCV RNA and HCVcAg (R^2^ = 0.8, p<0.0001). The median HCV RNA was 5.4 log IU/mL (95% CI: 4.99 to 5.71 IU/mL), and the median HCVcAg concentration was 2.5 log fmol/L (95% CI: 2.11 to 2.7) **(Fig 4)**.

**Fig 5:**
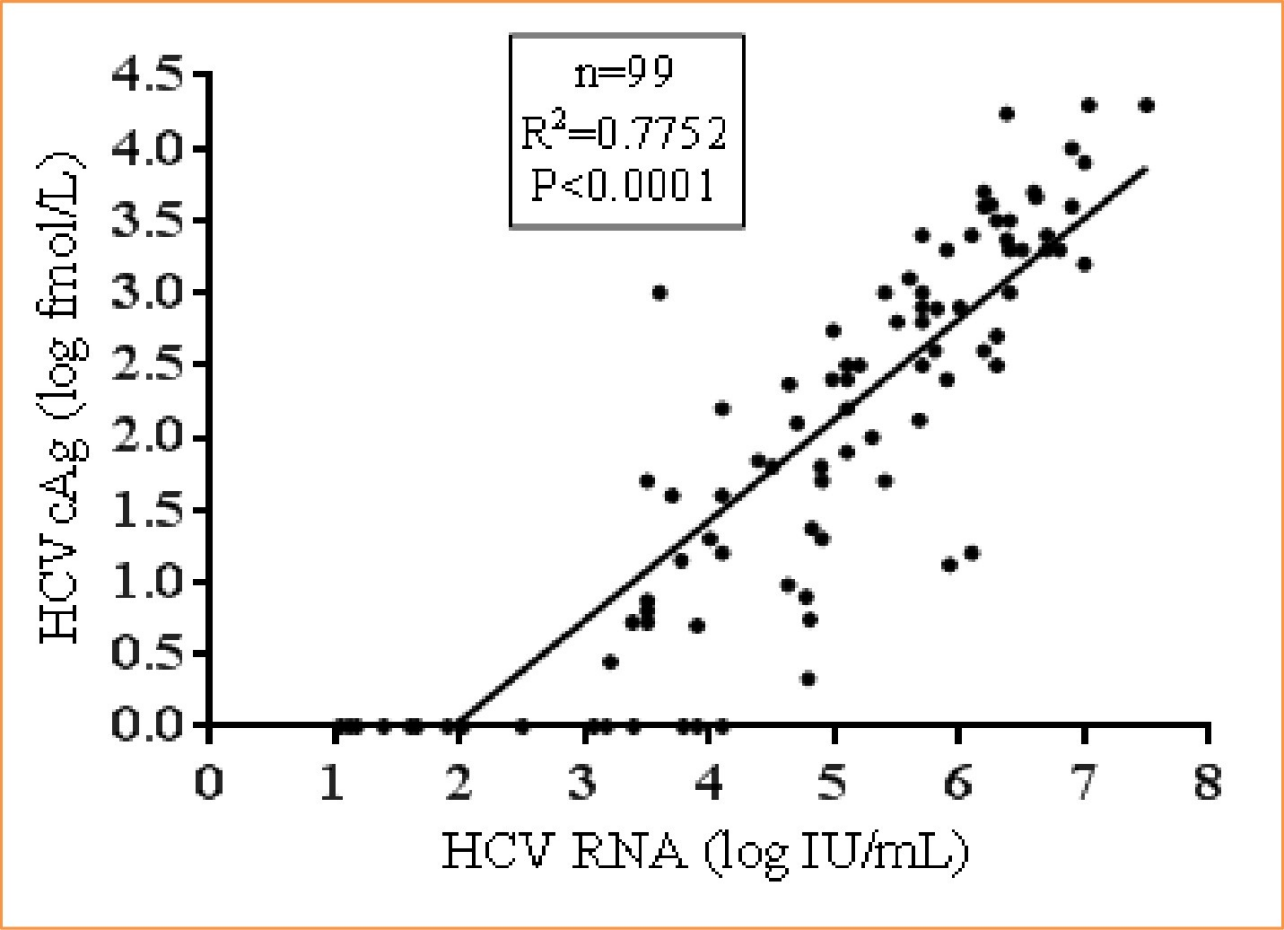
Correlation between HCV RNA and HCVcAg in HCV RNA and anti-HCV positive samples.

In samples (n = 99) that were positive for both HCV RNA and anti-HCV, a good correlation was found between HCV RNA and HCVcAg (R^2^ = 0.77, p<0.0001). The median HCV RNA was 5.1 log IU/mL (95% CI: 4.79 to 5.69 IU/mL), and the median HCVcAg concentration was 2.2 log fmol/L (95% CI: 1.7 to 2.57) **(Fig 5)**.

**Fig 6:**
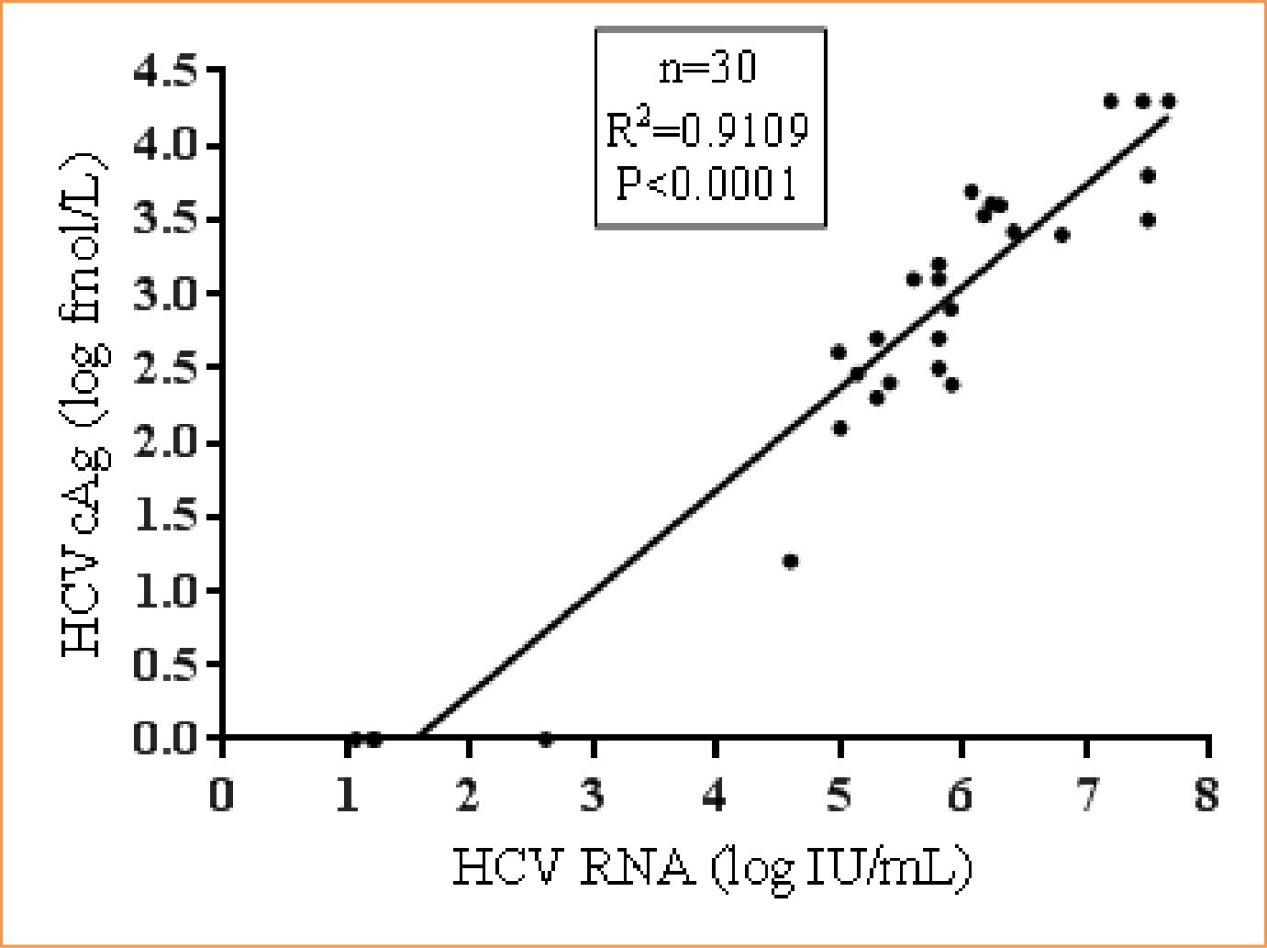
Correlation between HCV RNA and HCVcAg in HCV RNA positive with anti-HCV negative samples.

In samples (n = 30) that were positive for HCV RNA and negative for anti-HCV, an excellent correlation was found between HCV RNA and HCVcAg (R^2^ = 0.91, p<0.0001). The median HCV RNA was 5.8 log IU/mL (95% CI: 5.31 to 6.15 IU/mL), and the median HCVcAg concentration was 3 log fmol/L (95% CI: 2.46 to 3.41) **(Fig 6)**.

### Correlation between HCV RNA and HCVcAg in common genotype samples

**Fig 7:**
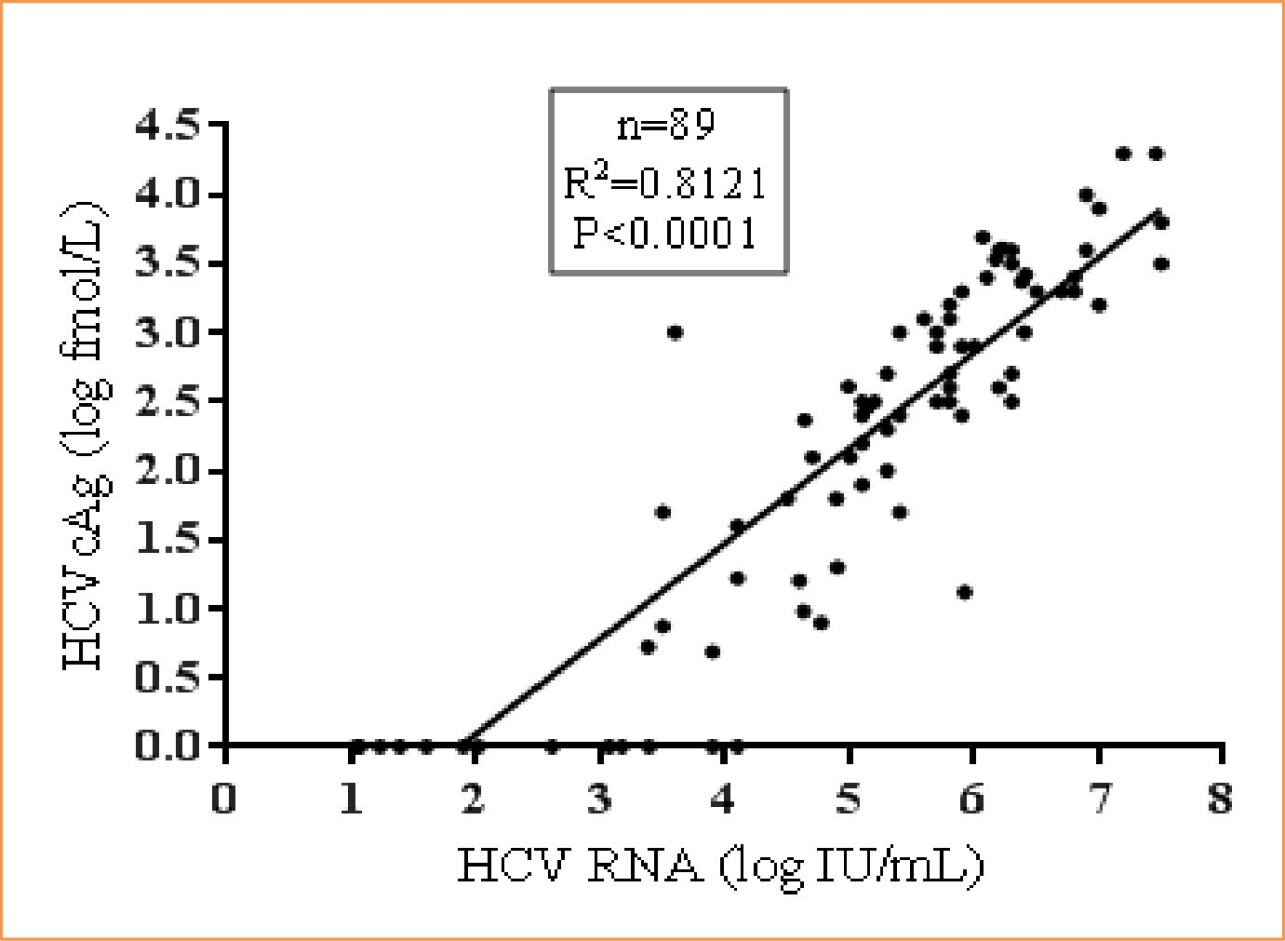
Correlation between HCV RNA and HCVcAg in common genotype samples.

Samples (n = 89) with three common genotypes (1, 3 & 4) showed good correlation between HCV RNA and HCVcAg (R^2^ = 0.81, p<0.0001). The median HCV RNA was 5.4 log IU/mL (95% CI: 5.1 to 5.8 IU/mL), and the median HCVcAg concentration was 2.5 log fmol/L (95% CI: 2.27 to 2.7) **(Fig 7)**.

### Receiver operating characteristic curve (ROC) analysis

**Fig 8:**
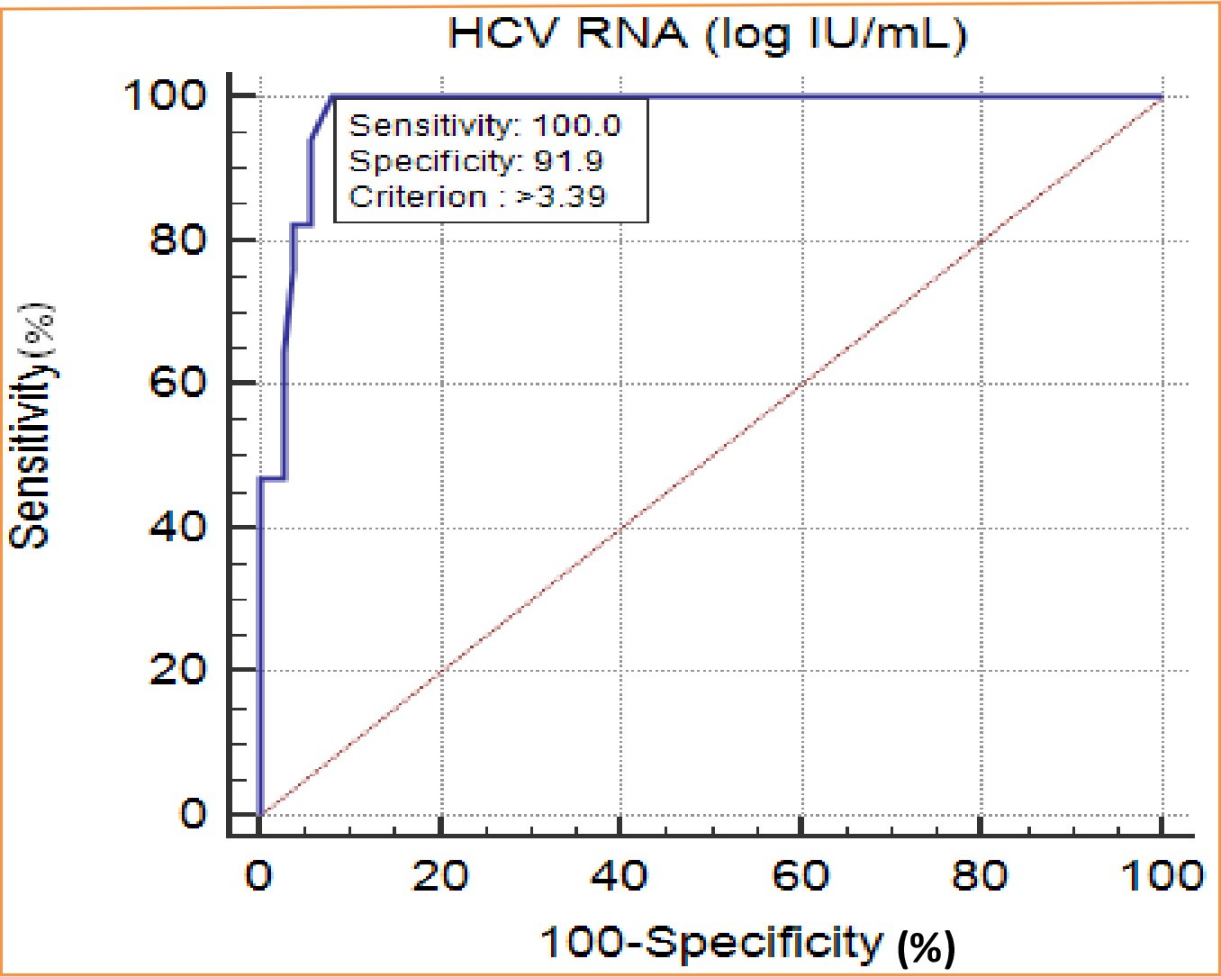
Receiver operating characteristic curve (ROC) analysis.

The receiver operating characteristic curve provided the sensitivity of 100% and the specificity of 91.9% with the viral load of >3.39 log IU/mL, which is equivalent to 2500 IU/mL of HCV RNA **(Fig 8)**.

## Discussion

HCVcAg and HCV RNA are the direct markers of HCV replication. However, there is a paucity of data on the utility of HCVcAg in hemodialysis and renal transplant patients. Such patients have a higher risk of HCV acquisition associated with disease management [5]. As the required frequency of HCV RNA testing is higher in this group, molecular testing escalates the overall cost by threefold when compared to the direct HCVcAg testing. Also, the reliability of anti-HCV antibody screening is not optimum among hemodialysis and renal transplant patients due to immunosuppression [17]. Hence, this study ascertained the utility of HCVcAg as an affordable viral marker to diagnose active HCV infection.

Samples for HCV RNA quantification are required to be frozen until the time of testing. Serum or plasma samples for HCVcAg do not require such specimen handling. This also makes it easier transportation to the nearest laboratory for HCVcAg estimation, and it can be tested on the same automated platform used for anti-HCV antibodies. The high positive predictive value of this HCVcAg permits screening of CKD patients before they start dialysis in a clinical setting. Due to the ease of testing and faster turnaround time, this assay can rapidly segregate infected and uninfected HCV patients.

This study demonstrated that the overall reliability and comparability of the HCVcAg with HCV RNA is good (R^2^ = 0.86, p<0.0001). Comparing the HCVcAg assay with an entire range of HCV viral loads (1 to 7 log IU/mL) representing the natural course of HCV infection yielded ≥ 80% sensitivity. Analysis of HCV viral load data excluding ≤3 log IU/ml markedly improved the overall sensitivity to 99% (data not shown), which is concordant with global data [18]. Typically in treatment naïve individuals, HCV viral load is in the range of 10^5^ to 10^6^ IU/mL [19]. A large cohort study (n = 10006) reaffirmed the clinical utility of HCVcAg as a screening/diagnostic assay to identify active HCV infection [20]. Furthermore, current study data is concordant with EASL (2016) and the WHO Global Hepatitis Report (2017) recommendations to implement HCVcAg to identify active HCV infection [21,22].

In anti-HCV negative with HCV RNA positive samples (n = 30), the HCVcAg assay showed an excellent correlation with HCV RNA (R^2^ = 0.91). This can be attributed to the lack of measurable humoral response resulting in a higher HCV viral load than the anti-HCV positive patients. As the CKD group has a higher preponderance for immunosuppression leading to anti-HCV negative status, HCVcAg adds important diagnostic value in reliably identifying active HCV infection. Hence, this cost-effective assay with comparable performance characteristics with HCV RNA can be considered to identify active HCV infection.

A reliable direct HCV detection assay should be able to identify active infection caused by common genotypes. The current HCVcAg assay showed a good correlation with HCV RNA in hemodialysis and renal transplant patients infected with common genotypes (1, 3 & 4). However, the reliability of the assay needs further evaluation with other HCV genotypes.

One of the limitations of the study finding is the reduced sensitivity of HCVcAg assay with low-level HCV RNA samples, which showed a divergence of 10^3^ IU/mL. This discordance is expected because intrinsically PCR-based target amplification techniques are much more sensitive than signal amplification serological techniques [23]. To overcome this addition of polyethylene glycol (PEG) may be used during the HCVcAg assay which is a potentiator of Ag-Ab reactivity. This is achieved by steric exclusion of Ag and Ab by the PEG being more exposed in the dispersion medium and facilitating the Ag-Ab interaction [24]. HCV infection elicits strong anti-HCV core-antibodies, and there is a plausibility of HCVcAg and core-Ab complexes compromising the sensitivity of the HCVcAg assay cannot be ignored. Thus, dissociation of HCVcAg from anti-HCV antibodies may enhance the sensitivity of HCVcAg assay, which requires careful validation [25,26].

## Conclusions

HCVcAg is a sensitive and specific direct viral marker of active HCV replication. This assay is logistically easier, cost-effective, easy to perform, reproducible with a shorter turnaround time than HCV RNA testing. In low-resource settings, HCVcAg can be used as a confirmatory marker to replace HCV RNA testing in detecting active HCV infection among hemodialysis and renal transplant patients. In HCVcAg negative cases, sequential testing with anti-HCV antibody followed by HCV RNA could be a reliable and cost-effective approach. The clinical utility of this assay can be confidently extended to hemodialysis and renal transplant patients.

## Supporting information

S1 File Overall data. (XLSX)

## References

[1] Mohd Hanafiah K, Groeger J, Flaxman AD, Wiersma ST. Global epidemiology of hepatitis C virus infection: new estimates of age-specific antibody to HCV seroprevalence. Hepatology. 2013;57: 1333–1342. 10.1002/hep.26141 23172780

[2] Goodkin DA, Bieber B, Jadoul M, Martin P, Kanda E, Pisoni RL. Mortality, Hospitalization, and Quality of Life among Patients with Hepatitis C Infection on Hemodialysis. Clin J Am Soc Nephrol CJASN. 2017;12: 287–297. 10.2215/CJN.07940716 27908905 PMC5293341

[3] Morales JM, Fabrizi F. Hepatitis C and its impact on renal transplantation. Nat Rev Nephrol. 2015;11: 172–182. 10.1038/nrneph.2015.5 25643666

[4] Agarwal SK, Dash SC, Irshad M. Hepatitis C virus infection during haemodialysis in India. J Assoc Physicians India. 1999;47: 1139–1143. 11225211

[5] Jasuja S, Gupta AK, Choudhry R, Kher V, Aggarwal DK, Mishra A, et al. Prevalence and associations of hepatitis C viremia in hemodialysis patients at a tertiary care hospital. Indian J Nephrol. 2009;19: 62–67. 10.4103/0971-4065.53324 20368926 PMC2847810

[6] Fabrizi F, Messa P, Martin P. Health-related quality of life in dialysis patients with HCV infection. Int J Artif Organs. 2009;32: 473–481. 10.1177/039139880903200801 19844890

[7] Fabrizi F, Dixit V, Messa P. Impact of hepatitis C on survival in dialysis patients: a link with cardiovascular mortality? J Viral Hepat. 2012;19: 601–607. 10.1111/j.1365-2893.2012.01633.x 22863263

[8] Cruzado JM, Carrera M, Torras J, Grinyó JM. Hepatitis C virus infection and de novo glomerular lesions in renal allografts. Am J Transplant Off J Am Soc Transplant Am Soc Transpl Surg. 2001;1: 171–178. 12099366

[9] Fabrizi F, Messa P, Martin P, Takkouche B. Hepatitis C virus infection and post-transplant diabetes mellitus among renal transplant patients: a meta-analysis. Int J Artif Organs. 2008;31: 675–682. 10.1177/039139880803100801 18825640

[10] Li Cavoli G, Zagarrigo C, Schillaci O, Servillo F, Tralongo A, Coglitore M, et al. Hepatitis C Virus Core Antigen Test in Monitoring of Dialysis Patients. Hepat Res Treat. 2012;2012. 10.1155/2012/832021 23304475 PMC3523129

[11] El-Sherif A, Elbahrawy A, Aboelfotoh A, Abdelkarim M, Saied Mohammad A-G, Abdallah AM, et al. High false-negative rate of anti-HCV among Egyptian patients on regular hemodialysis. Hemodial Int Int Symp Home Hemodial. 2012;16: 420–427. 10.1111/j.1542-4758.2011.00662.x 22360424

[12] Kidney disease improving global outcomes. KDIGO clinical practice guidelines for the prevention, diagnosis, evaluation, and treatment of hepatitis C in chronic kidney disease. 2008. 10.1038/ki.2008.81 18382440

[13] Bruggmann P, Berg T, Øvrehus ALH, Moreno C, Brandão Mello CE, Roudot-Thoraval F, et al. Historical epidemiology of hepatitis C virus (HCV) in selected countries. J Viral Hepat. 2014;21 Suppl 1: 5–33. 10.1111/jvh.12247 24713004

[14] Mederacke I, Wedemeyer H, Ciesek S, Steinmann E, Raupach R, Wursthorn K, et al. Performance and clinical utility of a novel fully automated quantitative HCV-core antigen assay. J Clin Virol Off Publ Pan Am Soc Clin Virol. 2009;46: 210–215. 10.1016/j.jcv.2009.08.014 19766055

[15] Tanaka Y, Takagi K, Fujihara T, Kitsugi K, Fujiwara K, Hiramatsu K, et al. High stability of enzyme immunoassay for hepatitis C virus core antigen-evaluation before and after incubation at room temperature. Hepatol Res Off J Jpn Soc Hepatol. 2003;26: 261–267. 10.1016/s1386-6346(03)00113-x 12963424

[16] Daniel HD-J, David J, Raghuraman S, Gnanamony M, Chandy GM, Sridharan G, et al. Comparison of Three Different Hepatitis C Virus Genotyping Methods: 5′NCR PCR-RFLP, Core Type-Specific PCR, and NS5b Sequencing in a Tertiary Care Hospital in South India. J Clin Lab Anal. 2016;31. 10.1002/jcla.22045 27580956 PMC6816979

[17] Hanuka N, Sikuler E, Tovbin D, Mostoslavsky M, Hausman M, Orgel M, et al. Hepatitis C virus infection in renal failure patients in the absence of anti-hepatitis C virus antibodies. J Viral Hepat. 2002;9: 141–145. 10.1046/j.1365-2893.2002.00332.x 11876797

[18] Wasitthankasem R, Vichaiwattana P, Auphimai C, Siripon N, Klinfueng S, Tangkijvanich P, et al. HCV core antigen is an alternative marker to HCV RNA for evaluating active HCV infection: implications for improved diagnostic option in an era of affordable DAAs. PeerJ. 2017;5: e4008. 10.7717/peerj.4008 29134150 PMC5678506

[19] Heidrich B, Wiegand SB, Buggisch P, Hinrichsen H, Link R, Möller B, et al. Treatment of Naïve Patients with Chronic Hepatitis C Genotypes 2 and 3 with Pegylated Interferon Alpha and Ribavirin in a Real World Setting: Relevance for the New Era of DAA. PLOS ONE. 2014;9: e108751. 10.1371/journal.pone.0108751 25302676 PMC4193764

[20] Tilborg M van, Marzooqi SHA, Wong WWL, Maan R, Vermehren J, Maasoumy B, et al. HCV core antigen as an alternative to HCV RNA testing in the era of direct-acting antivirals: retrospective screening and diagnostic cohort studies. Lancet Gastroenterol Hepatol. 2018;3: 856–864. 10.1016/S2468-1253(18)30271-1 30274834

[21] EASL Recommendations on Treatment of Hepatitis C 2016. J Hepatol. 2017;66: 153–194. 10.1016/j.jhep.2016.09.001 27667367

[22] WHO | Global hepatitis report, 2017. World Health Organization; Available: http://www.who.int/hepatitis/publications/global-hepatitis-report2017/en/.

[23] Mackay IM, Arden KE, Nitsche A. Real-time PCR in virology. Nucleic Acids Res. 2002;30: 1292–1305. 10.1093/nar/30.6.1292 11884626 PMC101343

[24] Garcia VS, Gonzalez VDG, Marcipar IS, Vega JR, Gugliotta LM. Optimisation and standardisation of an immunoagglutination assay for the diagnosis of Trypanosoma cruzi infection based on latex-(recombinant antigen) complexes. Trop Med Int Health TM IH. 2014;19: 37–46. 10.1111/tmi.12225 24219561

[25] Laperche S, Le Marrec N, Simon N, Bouchardeau F, Defer C, Maniez-Montreuil M, et al. A new HCV core antigen assay based on disassociation of immune complexes: an alternative to molecular biology in the diagnosis of early HCV infection. Transfusion (Paris). 2003;43: 958–962. 10.1046/j.1537-2995.2003.00430.x 12823757

[26] Daniel HDJ, Vivekanandan P, Raghuraman S, Sridharan G, Chandy GM, Abraham P. Significance of the hepatitis C virus (HCV) core antigen as an alternative plasma marker of active HCV infection. Indian J Med Microbiol. 2007;25: 37–42. 10.4103/0255-0857.31060 17377351

