# Supplementary material for: Clinical utility of hepatitis C virus core antigen (HCVcAg) assay to identify active HCV infection in hemodialysis and renal transplant patients
Source: PLoS One. 2021 Apr 22;16(4):e0250263. doi: 10.1371/journal.pone.0250263 (PMC8061997; doi:10.1371/journal.pone.0250263)
Supplement: Attachment — Submitted filename: Response to reviewers.docx [file pone.0250263.s002.docx]

**Journal requirements:**

1. Please ensure that your manuscript meets PLOS ONE's style requirements, including those for file naming. The PLOS ONE style templates can be found at

https://journals.plos.org/plosone/s/file?id=wjVg/PLOSOne_formatting_sample_main_body.pdf and https://journals.plos.org/plosone/s/file?id=ba62/PLOSOne_formatting_sample_title_authors_affiliations.pdf

**Ans: The contents of the manuscript modified according to the PLOS ONE requirements.**

2. Please include your tables as part of your main manuscript and remove the individual files. Please note that supplementary tables (should remain/ be uploaded) as separate "supporting information" files.

**Ans: Table included in the main part of the manuscript and uploaded its supporting information file**

3. We note that you have included the phrase “data not shown” in your manuscript. Unfortunately, this does not meet our data sharing requirements. PLOS does not permit references to inaccessible data. We require that authors provide all relevant data within the paper, Supporting Information files, or in an acceptable, public repository. Please add a citation to support this phrase or upload the data that corresponds with these findings to a stable repository (such as Figshare or Dryad) and provide and URLs, DOIs, or accession numbers that may be used to access these data. Or, if the data are not a core part of the research being presented in your study, we ask that you remove the phrase that refers to these data.

**Ans: All relevant data are within the manuscript and uploaded its supporting information file.**

4. Your ethics statement should only appear in the Methods section of your manuscript. If your ethics statement is written in any section besides the Methods, please delete it from any other section.

**Ans: The ethics statement appeared only in the methodology of the manuscript and it is removed from the other section.**

5. Please include a separate caption for each figure in your manuscript.

**Ans: Separate caption for each figure has been included in the manuscript.**

Reviewers' comments:

Reviewer's Responses to Questions:

**Comments to the Author**

1. Is the manuscript technically sound, and do the data support the conclusions?

The manuscript must describe a technically sound piece of scientific research with data that supports the conclusions. Experiments must have been conducted rigorously, with appropriate controls, replication, and sample sizes. The conclusions must be drawn appropriately based on the data presented.

Reviewer #1: Partly

Reviewer #2: Partly

**Response:**

**Assay calibration was performed followed by kit controls were included in everyday of run for HCV antibody testing, HCV RNA and HCVcAg quantification. In this study totally 231 samples from 140 patients were collected and tested. Samples with weak positive for anti-HCV and HCVcAg were repeated in duplicates as per the recommendations of testing manual.**

**Anti-HCV antibody testing: Kit calibrators, kit controls (negative & positive control)HCV RNA quantification: Kit calibrators, kit controls (negative, low positive & high positive control) HCVcAg quantification: Kit calibrators, kit controls (negative, low positive & high positive control)**

2. Has the statistical analysis been performed appropriately and rigorously?

Reviewer #1: Yes

Reviewer #2: Yes

3. Have the authors made all data underlying the findings in their manuscript fully available?

The [PLOS Data policy](http://www.plosone.org/static/policies.action#sharing) requires authors to make all data underlying the findings described in their manuscript fully available without restriction, with rare exception (please refer to the Data Availability Statement in the manuscript PDF file). The data should be provided as part of the manuscript or its supporting information, or deposited to a public repository. For example, in addition to summary statistics, the data points behind means, medians and variance measures should be available. If there are restrictions on publicly sharing data—e.g. participant privacy or use of data from a third party—those must be specified.

Reviewer #1: No

Reviewer #2: Yes

**Response: All the relevant data are mentioned in the manuscript and uploaded its supporting information files.**

4. Is the manuscript presented in an intelligible fashion and written in standard English?

PLOS ONE does not copyedit accepted manuscripts, so the language in submitted articles must be clear, correct, and unambiguous. Any typographical or grammatical errors should be corrected at revision, so please note any specific errors here.

Reviewer #1: Yes

Reviewer #2: Yes

5. Review Comments to the Author

Please use the space provided to explain your answers to the questions above. You may also include additional comments for the author, including concerns about dual publication, research ethics, or publication ethics. (Please upload your review as an attachment if it exceeds 20,000 characters)

Reviewer #1: The authors investigated the clinical feasibility of the hepatitis C virus core antigen (HCVcAg) to detect active HCV infection in chronic kidney disease (CKD) pre/post-renal transplant patients. They recruited 231 plasma samples with predominant low HCV viral load and comparing HCVcAg testing with anti-HCV antibody and HCV RNA. They found that the specificity was 99% and sensitivity was 80.62% for HCVcAg comparing HCV RNA, and the PPV and NPV of HCVcAg were 99.1% and 80.2% respectively. They concluded that HCVcAg is a reliable and cost-effective marker in identifying active HCV infection in Indian CKD patients. Though not innovative, this is the first reported data applying HCVcAg on the detection of active HCV infection in pre/post renal transplant subjects in India. A few issues need further address or clarification for the version of this manuscript.

1. Clarify ‘CKD’ from ‘end-stage renal disease’ (ESRD) for this study population since pre/post-renal transplant including dialysis patients were enrolled and the authors did not give renal function data in the context and table 1.

**Ans: CKD is suitable term that can be used, because patients with CKD grade 4 (not on dialysis) also recruited along with end stage renal disease, hemodialysis, post renal transplant patients. Furthermore the renal function data has been included in the Fig.1 of the manuscript.**

2. How many patients received dialysis and what kind of dialysis (HD or CAPD) were they doing?

**Ans: 94/140 (67.14%) patients received hemodialysis therapy. All 94 patients received hemodialysis, wherein 11 patients went for renal transplantation. There were no patients with CAPD recruited in this cohort.**

3. Did HD patients have more anti-HCV and HCV RNA discrepancy in this cohort?

**Ans: Total no of samples collected from the 94 hemodialysis patients were 105 Among 105 samples, Anti-HCV positive with HCV RNA negative: 15 (14.28%)**

**Anti-HCV negative with HCV RNA positive: 8 (7.61%)**

4. How many patients had HCVcAg ‘weak positive’ especially in anti-HCV negative subjects in this cohort?

**Ans: There were no weak positive of HCVcAg especially in anti-HCV negative subjects in this cohort.**

5. Give serum creatinine or eGFR data in table 1 and re-organize the structure of table 1, e.g. give n (%) instead of n/143, rename HBV/HIV infected to HBV/HIV co-infected and give HBV vs. HIV patient numbers respectively.

**Ans: Table 1 has re-organized. Creatinine and eGFR profiles included separately in Fig.1**

Reviewer #2: 1. Title: To put as CKD is "misleading". In actual fact, the study cohort was dialysis & transplant patients.

**Ans: CKD is suitable term that can be used, because patients with CKD grade 4 with not on hemodialysis has also been recruited in the study.**

2. Introduction: Line 109, to say HCVcAg has comparable sensitivity as HCV RNA may not be fully accurate. Literature reported HCVcAg sensitivity is at best 90%.

**Response: HCVcAg has comparable sensitivity as HCV RNA only for 90% of the samples. (included in introduction line 103)**

3. Methodology: Line 146, it was not made clear by author if repeat testing was performed for sample which has values of 3 to 10fmol/l. This was recommended by the testing manual.

**Ans: Values between 3-10 fmol/L were repeated in duplicates as per the testing manual. Note: If both the values are negative it was considered as negative, whereas if one value is negative and the other value was positive, the sample result was considered as positive.**

4.Results:
a. The author only emphasised on the correlation between HCVcAg & HCV RNA. This is not good enough is clinical setting. The clinicians would be interested to know the performance of HCVcAg in term of false positive or false negative. We cannot afford to miss any HCV + cases, esp. in dialysis settings.

**Response: This is the first Indian study in which samples with entire spectrum of viral load levels were included. According to our study 80% of the samples can be identified active HCV infection by HCVcAg testing and the remaining 20% of low viral load samples can be tested for HCV RNA. To overcome this addition of Polyethylene glycol can be used to enhance the sensitivity of the HCVcAg assay.**

b. Based on this analysis, the sensitivity of anti-HCV was 79.2% (99 out of 129) & HCVcAg was 80% (104 out 129). The sensitivity of both tests was almost the same. The author did not further elaborate on how to resolve the 20% false negative cases for HCVcAg.

**Response: According to our study 80% of the samples can be identified active HCV infection by HCVcAg testing and the remaining 20% of the low viral load samples can be tested for HCV RNA. To overcome this addition of Polyethylene glycol can be used to enhance the sensitivity of HCVcAg assay.**

c. The author also did not report the Anti-HCV & HCVcAg testing results in HCV RNA negative samples (N=102). This is important to make sure there is no high false positive.

**Ans: HCV RNA negative samples (n=102) were divided into anti-HCV positive (n=58) and anti-HCV negative (n=44) groups. In the HCV RNA negative with anti-HCV positive group may reflects antiviral mediated or natural clearance.**

**1/102 (0.98%) sample had false positive for HCVcAg among HCV RNA negative samples.**

**(Please refer supplement information file.1)**

5. Conclusion: It was not clear that if the author is advocating using HCVcAg as screening test to replace Anti-HCV OR using HCVcAg to replace HCV RNA in detecting active HCV infection. If it is former, the author needs to discuss if performing HCV RNA in all HCVcAg negative cases to rule out the 20% false negative cases will be cost-effective. If it is later, the author needs to justify if HCVcAg has the comparable sensitivity & specificity to HCV RNA to be used as confirmatory test, based on this study results. Sequential testing with Anti-HCV, followed by HCVcAg & HCV RNA only in HCVcAg negative cases will be a potential cost-effective approach to explore.

**Response: According to our study 80% of the samples can be identified for active HCV infection by HCVcAg testing and the remaining 20% of low viral load samples can be tested for HCV RNA. As you said sequential testing of anti-HCV followed by HCVcAg & HCV RNA in HCVcAg negative cases could be a potential cost-effective approach.**

6. PLOS authors have the option to publish the peer review history of their article ([what does this mean?](https://journals.plos.org/plosone/s/editorial-and-peer-review-process#loc-peer-review-history)). If published, this will include your full peer review and any attached files.

If you choose “no”, your identity will remain anonymous but your review may still be made public.

**Do you want your identity to be public for this peer review?** For information about this choice, including consent withdrawal, please see our [Privacy Policy](https://www.plos.org/privacy-policy).

Reviewer #1: No

Reviewer #2: No
