# Supplementary material for: Clinical utility of hepatitis C virus core antigen (HCVcAg) assay to identify active HCV infection in hemodialysis and renal transplant patients
Source: PLoS One. 2021 Apr 22;16(4):e0250263. doi: 10.1371/journal.pone.0250263 (PMC8061997; doi:10.1371/journal.pone.0250263)
Supplement: Attachment — Submitted filename: Response to Revieweres.docx [file pone.0250263.s003.docx]

PONE-D-20-35848R1
Clinical utility of hepatitis C virus core antigen (HCVcAg) assay to identify active HCV infection in hemodialysis and renal transplant patients

**Journal requirements:**

Please review your reference list to ensure that it is complete and correct. If you have cited papers that have been retracted, please include the rationale for doing so in the manuscript text, or remove these references and replace them with relevant current references. Any changes to the reference list should be mentioned in the rebuttal letter that accompanies your revised manuscript. If you need to cite a retracted article, indicate the article’s retracted status in the References list and also include a citation and full reference for the retraction notice.

**Response: All the references were verified and modified as per the requirement of PLOS ONE.**

Reviewers' comments:

Reviewer's Responses to Questions

**Comments to the Author**

1. If the authors have adequately addressed your comments raised in a previous round of review and you feel that this manuscript is now acceptable for publication, you may indicate that here to bypass the “Comments to the Author” section, enter your conflict of interest statement in the “Confidential to Editor” section, and submit your "Accept" recommendation.

Reviewer #1: All comments have been addressed

Reviewer #2: (No Response)

2. Is the manuscript technically sound, and do the data support the conclusions?

The manuscript must describe a technically sound piece of scientific research with data that supports the conclusions. Experiments must have been conducted rigorously, with appropriate controls, replication, and sample sizes. The conclusions must be drawn appropriately based on the data presented.

Reviewer #1: Yes

Reviewer #2: Yes

3. Has the statistical analysis been performed appropriately and rigorously?

Reviewer #1: Yes

Reviewer #2: Yes

4. Have the authors made all data underlying the findings in their manuscript fully available?

The [PLOS Data policy](http://www.plosone.org/static/policies.action#sharing) requires authors to make all data underlying the findings described in their manuscript fully available without restriction, with rare exception (please refer to the Data Availability Statement in the manuscript PDF file). The data should be provided as part of the manuscript or its supporting information, or deposited to a public repository. For example, in addition to summary statistics, the data points behind means, medians and variance measures should be available. If there are restrictions on publicly sharing data—e.g. participant privacy or use of data from a third party—those must be specified.

Reviewer #1: Yes

Reviewer #2: Yes

5. Is the manuscript presented in an intelligible fashion and written in standard English?

PLOS ONE does not copyedit accepted manuscripts, so the language in submitted articles must be clear, correct, and unambiguous. Any typographical or grammatical errors should be corrected at revision, so please note any specific errors here.

Reviewer #1: Yes

Reviewer #2: Yes

6. Review Comments to the Author

Please use the space provided to explain your answers to the questions above. You may also include additional comments for the author, including concerns about dual publication, research ethics, or publication ethics. (Please upload your review as an attachment if it exceeds 20,000 characters)

Reviewer #1: The authors response to my comments in the revised manuscript point-by-point accordingly though we have different opinion on definition of the study population (only CKD stage 4 & 5 were recruited in this study), I have no further comments.

**Response: We concur with your comment. As per your specific suggestion we have changed the definition of the study population as hemodialysis and renal transplant patients. This is included in the study title and manuscript.**

Reviewer #2: The authors have responded to the previous comments but some are not reflected in the revised manuscript. I would like to highlight 2 comments which are not addressed satisfactorily in the revised manuscript as below:

1. "The author also did not report the Anti-HCV & HCVcAg testing results in HCV RNA
negative samples (N=102). This is important to make sure there is no high false positive."
- In my opinion, it is not good enough to put the data in supplementary document. I would like to see this to be reported in main manuscript. It should be made clear to readers that HCV core Ag does not have high rate of false positive.

**Response: Classification of samples based on assay results has included in the main part of the manuscript and Figure 2.**

2. "It was not clear that if the author is advocating using HCVcAg as screening test to
replace Anti-HCV OR using HCVcAg to replace HCV RNA in detecting active HCV infection.
If it is former, the author needs to discuss if performing HCV RNA in all HCVcAg negative
cases to rule out the 20% false negative cases will be cost-effective. If it is later, the author needs
to justify if HCVcAg has the comparable sensitivity & specificity to HCV RNA to be used as
confirmatory test, based on this study results. Sequential testing with Anti-HCV, followed by
HCVcAg & HCV RNA only in HCVcAg negative cases will be a potential cost-effective
approach to explore."
- The concern on how to handle the 20% false negative cases was not addressed in the revised manuscript even though the authors responded to the comments. In the conclusion, the readers can only get the message from the authors that "In low-resource settings, HCVcAg can be used as a confirmatory marker to replace HCV RNA testing in detecting active HCV infection." If authors agree that sequential testing with Anti-HCV, followed by HCVcAg & HCV RNA only in HCVcAg negative cases is a potential approach, this should be discussed in the manuscript.

**Response: According to our study 80% of the samples can be identified for active HCV infection by HCVcAg testing and the remaining 20% of low viral load samples can be tested for HCV RNA. As per your valid suggestion, sequential testing with anti-HCV followed by HCVcAg & HCV RNA in HCVcAg negative cases will be a reliable and cost-effective approach. This is included in the conclusion part of the manuscript.**

7. PLOS authors have the option to publish the peer review history of their article ([what does this mean?](https://journals.plos.org/plosone/s/editorial-and-peer-review-process#loc-peer-review-history)). If published, this will include your full peer review and any attached files.

If you choose “no”, your identity will remain anonymous but your review may still be made public.

**Do you want your identity to be public for this peer review?** For information about this choice, including consent withdrawal, please see our [Privacy Policy](https://www.plos.org/privacy-policy).

Reviewer #1: No

Reviewer #2: No

[NOTE: If reviewer comments were submitted as an attachment file, they will be attached to this email and accessible via the submission site. Please log into your account, locate the manuscript record, and check for the action link "View Attachments". If this link does not appear, there are no attachment files.]

While revising your submission, please upload your figure files to the Preflight Analysis and Conversion Engine (PACE) digital diagnostic tool, <https://pacev2.apexcovantage.com/>. PACE helps ensure that figures meet PLOS requirements. To use PACE, you must first register as a user. Registration is free. Then, login and navigate to the UPLOAD tab, where you will find detailed instructions on how to use the tool. If you encounter any issues or have any questions when using PACE, please email PLOS at [figures@plos.org](mailto:figures@plos.org). Please note that Supporting Information files do not need this step.
